# Stem Cell Therapy for Cirrhosis of Liver in Bangladesh: Specific Design Compatible for Developing Country

**DOI:** 10.5005/jp-journals-10018-1277

**Published:** 2019-02-01

**Authors:** Mamun Al Mahtab, Sheikh MF Akbar, Masuda Begum, Md. A Islam, Md. A Rahim, Sheikh M Noor-E-Alam, Md. A Alam, Faiz A Khondaker, Ahmed L Moben, Masuda Mohsena, Md. Sakirul I Khan, Md. Z Huq, Swati Munshi, Ashraful Hoque, Sheikh A Haque

**Affiliations:** 1 Department of Hepatology, Bangabandhu Sheikh Mujib Medical University, Dhaka, Bangladesh; 2 Department of Pathology, Ehime University Proteo-Science Center, Ehime University Graduate School of Medicine, Ehime, Japan; 3 Miyakawa Memorial Research Foundation, Tokyo, Japan; 4 Department of Haematology, Bangabandhu Sheikh Mujib Medical University, Dhaka, Bangladesh; 5 Department of Transfusion Medicine, Bangabandhu Sheikh Mujib Medical University, Dhaka, Bangladesh; 6 Department of Hepatology, Abdul Malek Ukil Medical College, Noakhali, Bangladesh; 7 Department of Hepatology, M. Abdur Rahim Medical College, Dinajpur, Bangladesh; 8 Department of Hepatology, Shaheed Suhrawardy Medical College, Dhaka, Bangladesh; 9 Department of Medicine, Kurmitola General Hospital, Dhaka, Bangladesh; 10 Department of Community Medicine, Ibrahim Medical College, Dhaka, Bangladesh; 11 Department of Anatomy and Embryology, Ehime University Graduate School of Medicine, Ehime, Japan; 12 Department of Anesthesiology, National Institute of Cardiovascular Diseases, Dhaka, Bangladesh; 13 Department of Radiology and Imaging, Square Hospital Ltd., Dhaka, Bangladesh; 14 Centre for Medical Biotechnology, Directorate General of Health Services, Dhaka, Bangladesh; 15 Department of Transfusion Medicine, Impulse Hospital, Dhaka, Bangladesh

**Keywords:** Ascites, Decompensated, Liver cirrhosis, Stem cell

## Abstract

**Aims and objectives:**

To assess the safety and efficacy of stem cell therapy in patients with cirrhosis of the liver (LC) in the context of developing country with limited facilities for cell-based therapy and advanced technologies.

**Materials and methods:**

A total of 34 patients received granulocyte colony-stimulating factor at a dose of 30 IU, daily for 2 to 11 days to upregulate the numbers of white blood cells and stem cells. Subsequently, stem cells were isolated from the peripheral blood of LC patients in a closed chamber using a harvesting machine. Variable amounts of autologous stem cells were injected to LC patients for once. The patients were followed for 3 months and various factors related to safety and parameters of efficacy were analyzed in this interim report.

**Results:**

Out of 34 patients available for final analysis, 3 months after the start of stem therapy, 4 patients died within this period. There was no significant alteration in biochemical parameters due to stem cell therapy, and patients also did not develop any features of acute liver failure indicating that short-term safety parameters of stem cell therapy may be acceptable. Stem cell therapy had a dominant effect on ascites of in this cohort. Although 24 of 34 patients had ascites at the start of therapy,ascites were found in 11 patients after one month and only 4 patients had ascites after 3 months. The positive role of stem cell therapy on ascites in LC patients may be attributed, even in part, to increased serum levels of albumin after therapy compared to basal levels (p <0.001).

**Conclusion:**

This first study about stem cell therapy in Bangladesh indicates that cell therapy may be accomplished in general hospitals of developing countries if the proper design and mild to moderate types of invasive approach is utilized. The apparent safety of administered stem cells in LC patients and the observed effect on ascites of LC patients inspire optimism about the installation of new and innovative therapy in Bangladesh. Future studies with phase I/II may with stem cell and others cell may be planned at Bangladesh in patients with LC and other intractable diseases with suitable control arms.

**How to cite this article:** Mahtab MA, Akbar SMF, Begum M, Islam MA, Rahim MA, Noor-E-Alam SM, Alam MA, Khondaker FA, Moben AL, Mohsena M, Khan SI, Huq MZ, Munshi S, Hoque A, Haque SA. Stem Cell Therapy for Cirrhosis of Liver in Bangladesh: Specific Design Compatible for Developing Country. Euroasian J Hepatogastroenterol, 2018;8(2):121-125.

## INTRODUCTION

Billions to millions of human beingshave been infected with hepatitis viruses capable of inducing of chronic liver diseases. Additionally, an undefined number of patientsare prone to develop chronic liver diseases due to several other non-viral etiologies. Thus, about 500 million people are at risk of developing a complication of chronic liver diseases such ascirrhosis of the liver (LC).^1,2^ In LC, the hepatic lobular structure is destroyed with distortion of hepatic hemodynamics making them incapable to accomplish several important vital functionsof essential for our daily life. Once a person develops LC, it has long been regarded as an irreversible pathological process and medical management of LC is directed to symptomatic management only. Recently, it has been realized that LC may not be a completely irreversible pathological process and there remains an option to get a functional liver even after developing LC.^3,4^ Although various “School of Thoughts” have been prevailing for shaping a diseased liver, the most rationale one may be to induce a process of regeneration of healthy hepatocytes in diseased liver of LC patients; a fact that represents a process that can be accomplished by cell therapy, particularly by stem cell therapy.^5,6^

Traditionally, the objective of stem cell therapy in LC is to keep these patients in good shape so that they might get a new liver transplant. This is mainly a scenario of developed, advanced and rich countries.However, in developing countries that harbor more than 80% of LC patients, liver transplantation is not a practical therapeutic option.However, stem cell therapy may be evaluated if these can induce (a) improvement of the quality of life of LC patients, (b) delaying development of hepatocellular carcinoma (HCC), and (c) prolongation of survival with moderate levels of safety.

Bangladesh is the 7th most populous country of the world and harbors millions of patients with chronic liver diseases and tens of thousands of them have been developing LC. Most of these patients die with the extreme deterioration of the quality of life (QOL) with ascites, frequent variceal bleeding, andhepatic encephalopathy, and finally ending with liver failure or development of HCC. These bring tremendous economic and social burdens to the individuals and their families as well as to the health care delivery system of the country.

However, several sophisticated instruments and extremely safe Good Manufacturing Practices (GMP) levels facilities are required for stem cell therapy and many of these variables are not present in Bangladesh at this moment.

In this perspective, we planned a design of stem cell therapy that would ensure the proliferation or generation of stem cells by the action of growth factor endogenously in LC patients. Then, the isolation of stem cell was done in a closed chamber so that it remains free of contamination. Subsequent administrations of stem cells were also accomplished via a completely safe maneuver. These patients were followed up for 3 months after the end of stem cell therapy and some highly encouraging observations were reported. It is plausible that more sophisticated forms of stem cell therapy and other cell therapy may be accomplished in Bangladesh with careful consideration of all relevant facts. This first report of stem cell therapy has taught us the ways and means for accomplishing cell therapy and evolving therapy in developing countries with minimum to moderate facilities.

## MATERIALS AND METHODS

### Patients

Stem cell therapy was accomplished in 34 patients with LC; the diagnosis of each patient was accomplished by standard procedures based on the recommendations of international professional organizations like American Association for the Study of the Liver (AASLD), European Association for the Study of Liver (EASL) and Asia-Pacific Association for the Study of the Liver (APASL).^7-9^ The patents have been suffering from chronic liver diseases for a long time and developed LC over time. LC was diagnosed based on biochemical parameters and final confirmation was accomplished by imaging of liver and/ or liver biopsy. All of them were in a decompensated state with mostly ascites and esophageal varices. None of them had HCC. Also, patients had no other concurrent diseases that may influence host immunity other than LC. Patients were not taking any immune modulators at the time clinical trial.

### Standard of Care Therapy for LC at Bangabandhu Sheikh Mujib Medical University; the Study Site

In this study, all patients received standard care therapy for LC. The study was conducted at the Bangabandhu Sheikh Mujib Medical University (BSMMU), the only medical university of Bangladesh. The patients with LC, especially those with decompensated LC can be offered in the most efficient manner the standard care therapy at BSMMU. Unfortunately, all patients with LC those deserve admission and therapy may not be admitted to BSMMU due to the limitation of beds for the patients. Besides, there is no national health insurance system in Bangladesh. Thus, many patients cannot buy many ailments of the standard of cure therapy for LC. We enrolled patients serially after obtaining informed consent and all aspiring patients were enrolled for study with no exclusion at the intention to treat (ITT) level.

At BSMMU, the standard of care therapy was characterized by two main principles: (1) treatment of etiology by antiviral drugs when infection with HBV or HCV can be affirmed; (2) symptomatic treatment of the patients: human albumin is given for substitution of low albumin; Bowel care is taken by lactulose. In the case of varices and variceal bleeding, all recommended therapies are employed based on the conditions of the patient and recommendations of the medical board.

### Pretreatment Administration of Granulocyte Colony Stimulating Factor (GCSF) to Accentuate the Frequencies of Stem Cell

All patients receive pretreatment granulocyte colony stimulating factor (GCSF) (30IU) daily for 2 to 11 days around the umbilicus. The safety of the patients after administration of GCSF was properly evaluated and when there was no concern about GCSF-induced safety, the patients were provisionally selected for stem cell therapy.

### Stem Cell Harvesting from Patients

Autologous stem cells were used for the treatment of these patients in addition to standard care therapy for LC. Stem cell harvesting has been done after 2 to 11 daily administration of GCSF. Stem cell was isolated based on a close chamber-related isolation method, exactly as described by the manufacturer and optimized by us. The apheresis machine (COM.TEC, Fresenius Kabi AG, Hamburg, Germany) was used to get the enriched population of stem cells and this minimized the possibility of contamination. A commercial kit called P1YA kit (Fresenius Kabi AG, Hamburg, Germany) has been used for harvesting stem cells from peripheral blood. After counting and confirming the viability of stem cells, these were ready for administration to LC patients.

A catheter was inserted into the femoral vein. Acid citrate dextrose (ACD)-1 (Fresenius Kabi AG,) has usually been used as an anticoagulant during a procedure at a ratio of 12-14:1. Injection with calcium gluconate (10%) was given to counter the adverse reaction of ACD-1. The patient was under continuous monitoring by cardiac monitor throughout the procedure. The machine is a continuous type of apheretic machine where blood is collected and undergo centrifugation for the collection of stem cells. At the same time, other types of blood cells and plasma are returned to the circulation of the patient. Approximately 60 to 65 mL stem cells have been collected and the numbers of stem cells were calculated by flow cytometry (Bacton Dickenson FACSVerse,Bacton Dickenson Biosciences, San Jose, CA, USA). Stem cells were administered by intravenous route in all patients on this cohort.

### Follow-up of the Patients

The procedures of follow-up of the patients were carefully planned based on various factors such as socioeconomic status, clinical limitations of developing countries, and reflecting a real-life situation for this type of therapy in future. The initial follow-up was planned for after 3 months and then secondary follow up will be accomplished after 6 and 12 months. During the initial follow up of 3 months, general features of the patients were analyzed. Also, the parameters of liver and kidney functions were assessed. Careful consideration was provided about the extent of ascites. However, the endoscopic analysis was not done routinely as that may be counterproductive in some patients.

### Statistical Analysis

Statistics were performed using the average values, and all data are reported as means ± SD (except mentioned elsewhere). The data on serum albumin level was statistically analyzed using the one-way repeated measurement of analysis of variance (ANOVA), and the Tukey-Kramer post-hoc comparisons. Data serum albumin levels are expressed as means ± SEM, and statistical significance was set at p < 0.001. The number of patients in each observation point is described in the Table 1.

## RESULTS

### Patient Profile

The mean age of the patient was 59 years (standard deviation, 10.87 years). There were 21 males and 13 females. The possible causes of LC were hepatitis B virus in 13 patients, hepatitis C virus in 4 patients, non-alcoholic steatohepatitis in 3 patients and alcoholic hepatitis in 3 patients. The etiological diagnosis could not be confirmed in 11 patients and they were termed as cryptogenic.

**Table 1:** **Table 1:** Biochemical parameters of patients with liver cirrhosis at different time points

		*Bilirubin*		*SGPT*		*Albumin*		*INR*		*PT*		*Creatinine*	
Basal		2.2 ± 2.5		38 ± 25		2.63 ± 0.45		1.67 ± 0.53		20.24 ± 6.63		1.09 ± 0.35	
One month after start of therapy		1.81 ± 1.55		35.55 ± 14.3		3.03 ± 0.48*		2.0 ± 2.2		18.41 ± 6.0		1.03 ± 0.36	
Three months after start of therapy		2.03 ± 1.80		33.0 ± 12.8		3.12 ± 0.39*		2.02±2.39		17.58±6.0		1.04±0.37	

SGPT-Serum glutamic-pyruvic transaminase; INR-International normalized ratio; PT-Prothrombin time p < 0.001

### Granulocyte Colony-Stimulating Factor in Positively Regulating Blood Cells

The GCSF induced increased numbers of WBC in all patients, although the effects of GCSF were highly variable among patients. The mean value of WBC was about 6200 cells/μl (range: 2200 to 13,000 cells/μl) before the start of administration of GCSF. After end of GCSF administration, the mean value of WBC raised to 26,000 cells/μl (12,000-54,000 cells/μl). The increase of WBC numbers by GCSF was not dependent on age or pathological status of the patients. Also, the etiological facts had no role on the effect of GCSF-induce cell modulation. Although 11 injections were required in one patient to have a considerable increase of WBC by GCSF, it seems that 3 to 6 injections with GCSF were enough to have doubling or tripling of WBC counts.

The number of stem cells isolated by close chamber procedure also varied considerably among patients. However, as a standard practice, we used at least 100,000 stem cells per patient. It is tobe mentioned that all patients also received standard of care therapy as practiced by Department of Hepatology of BSMMU, Dhaka, Bangladesh in addition to stem cell therapy.

### Survival Outcome

Although 38 patients were enrolled initially, we lost contact with 4 patients after enrollment. The data of these 4 patients have been excluded from the final analysis. A total of 4 patients died within the initial 3 months period of follow-up; two of them died within 1 month, 1 died within 2 months and the rest one died within 3 months. The cause of death was also variables those include heart failure, hepatic failure, and variceal bleeding.

### Effect of Stem Cell Therapy on Biochemical Parameters

**Graph 1:**
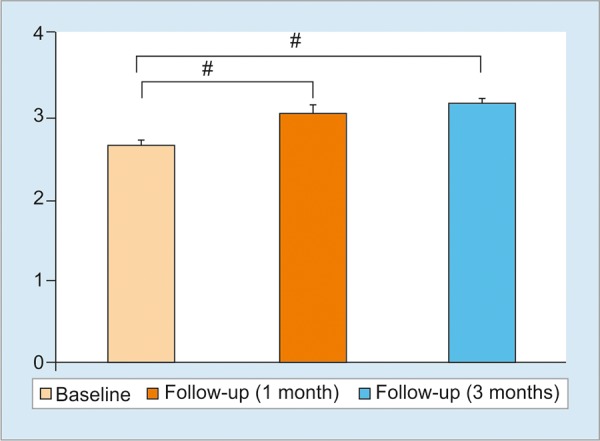
Levels albumin at different states before and after stem cell therapy in patients with liver cirrhosis

The biochemical data of the patients have been summarized in Table 1. Apparently, we did not find any significant change in the levels of serum bilirubin, ALT, AST, and PT before and at 1 and 3 months after therapy (Table 1). The only notable change by stem cell therapy was found in serum albumin level. The levels of albumin increased significantly one month after therapy compared to basal levels of serum albumin (p <0.001) (Graph 1). Also, the levels of albumin remain increased at the level of 1-month at 3 months after therapy.

### Ascites and Survival

Another dominant effect of stem cell therapy was recorded regarding ascites. At the start of therapy, 24 patients had ascites. After 1 month of stem cell therapy, it reduced to 11 patients and after 3 months, ascites was present in only 4 patients. All dead patients also had ascites.

## DISCUSSION

We have already used GCSF to activate the immune system in patients with liver failure and LC^10,11^ and retrieved moderate levels of effects of GCSF. Although the mechanisms underlying their therapeutic effect could not be properly evaluated due to technical limitations, therapy with GCSF induced better survival and quality of life in our cohort.

In the present communication, we used GCSF to increase the frequencies of hematopoietic stem cells in situ. These stem cells were isolated in a closed chamber. Finally, G-CSF-induced stem cells were administered to LC patients and their safety and efficacy were checked for 3 months. The approach seems to be safe as none developed acute flare of liver failure or other similar adverse reaction during treatment and 3 months of follow up period. Out of 34 patients that could be properly analyzed, 4 patients died within the follow-up period of 3 months. However, it is difficult to assess if the frequency of death at 11.8% (4 out of 34 patients with decompensated LC) was high or low as the study did not enroll a control group. The long-term clinical experiences of our team indicate that the death rate may be low using standard care of therapy plus stem cells compared to the only standard care of therapy. However, convincible data are required that should be evidence-based and a study is now in progress to evaluate this important issue.

Although the frequency of death of LC patients would remain as an issue, for the time being, extremely inspiring data evolved by analyzing the role of stem cell therapy on ascites of these patients. In this study, ascites was present in 24 of 34 patients at the basal level (before therapy) and this was seen only in 11 patients after one month and in 4 patients after 3 months of therapy. The most important feature is that the patient those got rid of ascites at one month did not develop ascites at 3 months. The factor responsible for the down-regulation of ascites was reflected in increased levels of albumin (Graph 1). This is a highly encouraging fact as ascites represent one of the most prominent factors related to the worst quality of life of LC patients. The real mechanisms underlying this is unknown, But, these patients also received human albumin, but usually, only human albumin does not improve the levels of albumin and ascites so drastically. It is tempting to conclude that a combination of human albumin plus stem cells may have a more potentiating action for containment of ascites and this should be checked in a large cohort with adequate power and controls. We used hemopoietic stem cells as these can be harvested safely in Bangladesh. However, other types of stem cells may be used in LC patients in the future.^12,13^

There may be multiple logics of the positive impact of cell therapy in LC patients. In one hand, investigators have mentioned that stem cell may be converted to healthy hepatocytes in the diseased liver. On the contrary, the immune modulatory effects of stem cell in LC liver may be beneficial to LC patients. However, this study was not designed to assess the conversion of infused stem cells as hepatocytes in the liver of LC patients due to the concern of safety and technical factors. The next, the stem cells were harvested in a closed chamber so that these may not be contaminated as good manufacturing practice (GMP) is yet to be introduced for cell therapy in Bangladesh.

## CONCLUSION

In conclusion, this is the first published approach of stem cell therapy in Bangladesh, although several investigators have focused on this issue in works of literature. It was very carefully designed to maintain the safety of the patients to their highest levels. Although stem cell therapy showed some survival benefit, this could not be highlighted properly as there has been a lack of control groups. However, the role of stem cell therapy on ascites seems to be a major contribution of this therapy. This helped to provide a better quality of life to LC patients. This study will also unmask various study with other types of stem cells for therapy. In this study, we administered stem cell for only in one cycle. This study indicates that stem cell therapy may be accomplished for several times with some time gap to have a better outcome. The dose and duration of therapy may also be important variables.
